# Sealing ability of Biodentine, zirconia reinforced glass ionomer cement and Mineral Trioxide Aggregate as furcation perforation repair materials: an *in vitro* analysis

**DOI:** 10.2340/biid.v13.45038

**Published:** 2026-01-15

**Authors:** Sumita Panwar, Yajuvender Singh Hada

**Affiliations:** Department of Conservative Dentistry and Endodontics, Daswani Dental College and Research Centre, Kota, Rajasthan, India

**Keywords:** Biodentine, Mineral Trioxide Aggregate angelus, Zirconia Reinforced Glass Ionomer cement, furcation perforation repair

## Abstract

**Aim:**

The present study aimed to evaluate and compare the sealing ability of Biodentine, zirconia reinforced glass ionomer cement (GIC), and Mineral Trioxide Aggregate (MTA) as furcation repair materials.

**Materials and methods:**

A total of 50 extracted permanent maxillary molars were collected and divided into three experimental groups and one control group. Group I – Biodentine (*n* = 15), Group II – zirconia reinforced GIC (*n* = 15), Group III – MTA Angelus (*n* = 15), and unrepaired, control group (*n* = 5). Crowns of teeth in experimental groups were sectioned 3 mm above the cementoenamel junction and roots 3 mm below the furcation. Standardised endodontic access openings were prepared, canal orifices and root ends were sealed with sticky wax. After coating with nail varnish, a 1 mm furcation perforation was created at a standardised location using a round carbide bur. Samples were flushed, dried, and incubated at 37°C for 24 h to simulate clinical conditions. All samples were subjected to orthograde and retrograde methylene blue dye challenge followed by dye extraction with a concentration of 65% nitric acid. Samples were then analysed using 550 ultraviolet-visible spectrophotometers.

**Statistical analysis:**

The results were analysed statistically by one-way analysis of variance (ANOVA) and Tukey’s multiple comparison tests.

**Result:**

No statistically significant difference in sealing ability was observed between Biodentine, zirconia reinforced GIC, and MTA when used as a furcation perforation repair material.

**Conclusion:**

Within the limitations of this study, it can be concluded that Biodentine, zirconia reinforced GIC, and MTA showed sealing ability comparable to each other.

## Introduction

The primary goal of endodontic therapy is to preserve the natural dentition while restoring its proper form, function, and aesthetics. However, procedural accidents frequently occur in endodontic practice, potentially compromising the success of root canal treatment. One such complication is a furcation perforation, which can significantly impact the prognosis, often leading to the most unfavourable treatment outcomes [1].

Ingle reported that root canal perforation is the second most common cause of endodontic failure and accounts for 9.6% of all unsuccessful cases. Perforations can be defined as mechanical or pathologic communications between the root canal system and the external tooth surface [2].

Misaligned use of rotary burs during endodontic access preparation and search for root canal orifices occurs in 2–12% of endodontically treated teeth [3]. Inappropriate post space preparation for permanent restoration of endodontically treated teeth is yet another common iatrogenic cause of root perforation [4]. Non-iatrogenic causes include root resorption and caries [5].

Once an infectious process has been established at the perforation site, the prognosis for treatment is precarious, and the complication may prompt extraction of the affected tooth [6]. For long-term success, perforations should be repaired as quickly as possible with a biocompatible material to prevent bacterial contamination [7]. The selection of materials with the appropriate qualities, such as biocompatibility, sealing ability, and tissue regeneration capability, is crucial for successful furcation perforation repair [8].

Several materials have been recommended for perforation repair, including zinc oxide eugenol cements (IRM and Super-EBA), glass ionomer cement (GIC), composite resins, resin-modified glass ionomer, amalgam, gutta-percha, calcium hydroxide, Cavit and, more recently, Mineral Trioxide Aggregate (MTA), Biodentine, and Endo Sequence root repair material. However, none of these materials fulfil the ideal requirements of a repair material [9].

Biodentine, a powder-liquid system, is a calcium silicate based bioactive material. . The powder is composed of tricalcium silicate, di-calcium silicate, calcium carbonate, iron oxide, and zirconium oxide, with the liquid consisting of calcium chloride and hydro-soluble polymer. It is easy to handle owing to its ease of manipulation and a short setting time (approximately 12 min). It has an alkaline pH and is a biocompatible material, making it a favourable material for perforation repair [10, 11].

Mineral Trioxide Aggregate (MTA) consists of fine hydrophilic particles of tricalcium silicate, tricalcium aluminate, tricalcium oxide, silicate oxide, calcium sulphate dihydrate, tetracalcium aluminoferrite, and small amounts of mineral oxides (bismuth oxide) [12]. MTA, the first calcium silicate-based bioactive endodontic cement (BEC), was introduced to the field of endodontics in the early 1990s as a root-end filling (REF) material. Due to the superior biological and clinical performance of MTA as compared to traditional materials, it soon emerged as the material of choice for several endodontic applications [13]. In this study, MTA Angelus was chosen because of its shorter setting time (~15 min) compared to conventional MTA (2–4 h).

Zirconomer (zirconia + GIC) is a recently launched glass ionomer formulation, designed to overcome the disadvantages of traditional GIC formulations. The powder consists of fluoroaluminosilicate glass, zirconium oxide, pigments, while the liquid contains a solution of polyacrylic acid and tartaric acid. It possesses the strength of amalgam, along with the beneficial effects of GICs, and eliminates the hazardous effects of mercury; hence, it is also referred to as white amalgam [14].

Dye extraction microleakage evaluation is a reliable and widely used method for assessing the sealing ability of dental materials. It provides a quantitative analysis by spectrophotometrically measuring the amount of dye that penetrates and is extracted from the material interface. The dye molecules are smaller than bacteria, allowing them to penetrate even the tiniest gaps; therefore, the presence of dye indicates potential pathways for bacterial leakage as well. This makes the method highly sensitive and clinically relevant. Compared to techniques such as fluid filtration or bacterial leakage tests, dye extraction is a more straightforward, cost-effective method that allows for an accurate comparison between materials in *in vitro* settings [15].

This study aimed to evaluate and compare the sealing ability of Biodentine, zirconia reinforced GIC, and MTA Angelus when used as furcation perforation repair materials using the spectrophotometric dye extraction method.

The null hypothesis was that there would be no significant difference in the sealing ability among the three tested materials.

## Materials and methodology

**Figure 1:**
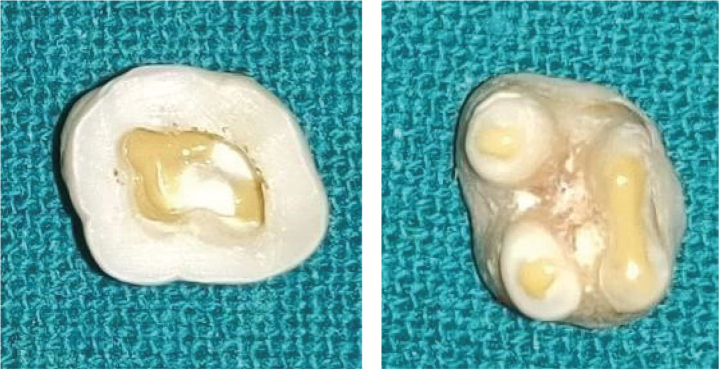
Decoronation 3 mm above the cementoenamel junction and 3 mm below the furcation. *Images for figures 1–6 used in this manuscript were self-captured by the author.

**Figure 2:**
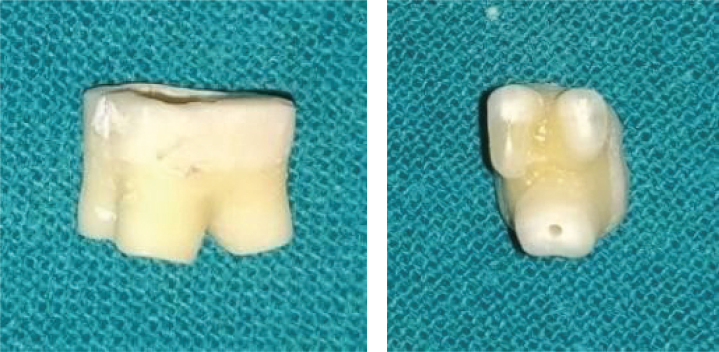
Sticky wax is placed on the canal orifices and root ends.

**Figure 3:**
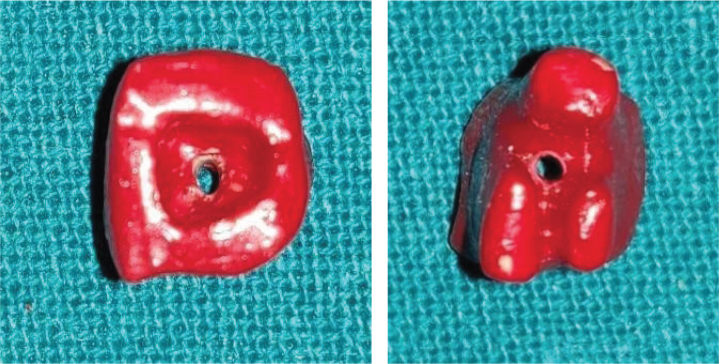
Perforation made in the centre of the furcation area.

A total of 50 caries-free and restoration-free human maxillary molars that were extracted due to unhealthy periodontal conditions were collected, stored, and disinfected according to the Occupational Safety and Health Administration (OSHA) regulations. Molars were decoronated 3 mm above the cemento-enamel junction, and roots were amputated 3 mm below the furcation after measuring with a periodontal probe with a diamond disk (Figure 1). A standardised endodontic access opening was prepared in all 50 samples. Sticky wax was placed over the orifice of each canal as well as on amputated roots (Figure 2). Teeth were then coated with two layers of nail varnish. To ensure each perforation was centred between the roots, a black marker pen was used to mark the location of the defect. A 1 mm in diameter defect was created on the external surface of the tooth using a number 2 round carbide bur in a handpiece with air and water coolant (Figure 3). The chamber and perforation were flushed with water and dried. The teeth were kept in an incubator at 37°C for 24 h to simulate clinical conditions.

**Graph 1:**
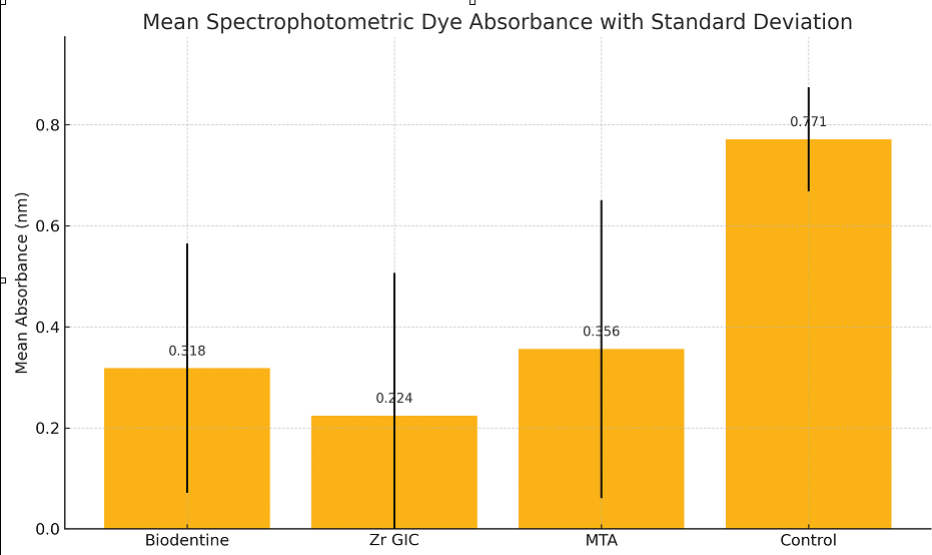
Mean spectrophotometric dye absorbance values of the groups. *Source*: Original.

### Perforation repair

**Figure 4:**
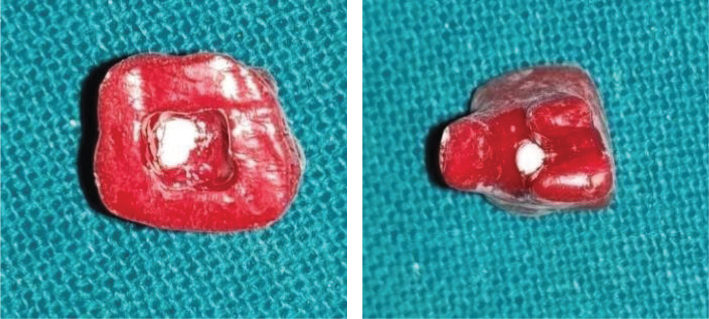
Perforation repair done with Biodentine.

Teeth were randomly divided into four groups with 15 samples in each of the three experimental groups and 5 samples in the control group. By using 15 samples per experimental group, the study increases power beyond the minimum requirement, reducing the risk of Type II error (false negatives) and enhancing the reliability of the results. The control group with five samples provides a baseline to compare leakage values, but may have less power for detecting subtle differences. However, since the control group showed significantly higher leakage, this sample size was sufficient to establish clear statistical differences. Three recent furcation perforation repair materials were used in the study (Table 1). These were: Group I: Teeth were repaired with Biodentine (Figure 4) (Septodont, Saint Maur des Foss’es, France), Group II: Teeth were repaired with zirconia reinforced GIC (Zirconomer, Shofu, Japan), Group III: Teeth were repaired with MTA (MTA Angelus, Angelus Odontolgia, Brazil). Group IV, that is, the control group, was left unrepaired (positive control). All the teeth in each group were left for 24 h to allow the materials to set.

**Table 1:** Materials studied.

Material	Composition	Manufacturer
Biodentine	Tricalcium silicate, calcium carbonate, zirconium oxide, additives in liquid (calcium chloride, water)	Septodont, France
Zirconia reinforced GIC Zirconomer	Glass ionomer cement with added zirconia particles, fluoroaluminosilicate glass, polyalkenoic acid	Shofu, Japan
Mineral Trioxide Aggregate MTA Angelus	Tricalcium silicate, dicalcium silicate, tricalcium aluminate, bismuth oxide (radiopacifier)	Angelus Dental, Brazil

## Dye extraction microleakage evaluation

**Figure 5:**
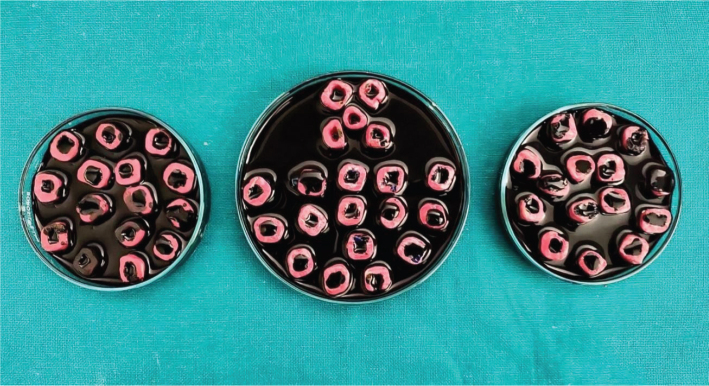
Each group was placed in a separate petri dish containing 2% methylene blue.

**Figure 6:**
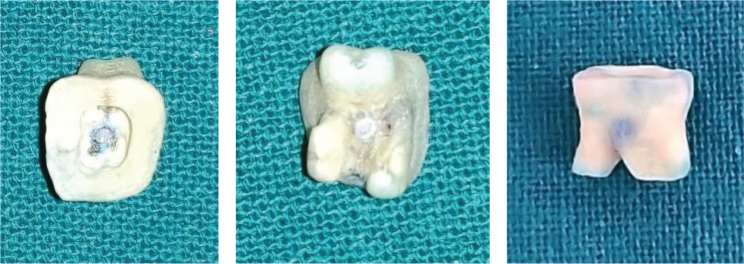
Varnish removed with a polishing disc.

Each group was placed in a separate Petri dish containing 2% methylene blue such that all teeth were immersed in dye up to the cemento-enamel junction for retrograde dye challenge, and dye was added to the access chamber of each tooth for orthograde dye challenge (Figure 5). All samples were stored for 48 h. After removal of the dye, teeth were rinsed under tap water for 30 min, followed by removal of the varnish with a polishing disc (Figure 6). Each tooth was stored in a vial containing 5 mL of concentrated nitric acid (65%) for 3 days. The solutions thus obtained were centrifuged at 3,500 rpm for 5 min in a centrifugal machine. Four mL of the supernatant liquid was then analysed in an ultraviolet (UV) visible spectrophotometer at 550 nm wavelength with concentrated nitric acid as the blank, and readings were recorded as absorbance units, which means the amount of dye penetration in each group. The obtained readings were statistically analysed using one-way analysis of variance (ANOVA) and Tukey’s HSD (Honestly Significant Difference) multiple comparisons tests.

To enhance the robustness of this study, additional physicochemical tests were included to evaluate material properties that may influence sealing ability. Thus, flow, setting time, and solubility of the three materials were measured.

The flow was conducted according to ISO 6876:2012 standards, where a set volume of freshly mixed material was placed between two glass plates under a standard load, and the diameter of the resulting disc was measured after 180 s.

The setting time was determined using a Gilmore needle apparatus under controlled temperature and humidity, in accordance with ISO 9917-1 for GICs and ISO 6876 for endodontic materials.

The solubility of the materials was determined following ISO 6876:2012 protocol, according to which disc specimens of each material were weighed before and after immersion in distilled water for 24 h at 37°C. The percentage weight loss was calculated.

These physicochemical properties were then analysed in context with the sealing ability results.

## Results

The mean absorbance values varied between 0.224 and 0.771 nm (Table 2). The values were found to vary with statistical significance (*p* = 0.003).

**Table 2:** Mean absorbance values (dye penetration; nm) for the four groups.

Groups (Materials)	Mean absorbance value (dye penetration) (nm)	Standard deviation (SD) (nm)	Standard error (SE) (nm)	Median (nm)	*F*	*p*
**Biodentine**	0.318	0.247	0.064	0.164	5.391	0.003**
**Zr GIC**	0.224	0.283	0.073	0.094		
**MTA**	0.356	0.295	0.076	0.139		
**Control**	0.771	0.103	0.046	0.761		

*Source*: Original.

MTA: Mineral Trioxide Aggregate.

**Highly statistically significant.

The results of the measurements of flow, setting time, and solubility are summarised in Table 3.

**Table 3:** Flow, setting time, and solubility of the materials evaluated.

Material	Flow (mm)	Setting Time (min)	Solubility (%)
Biodentine	20.5 ± 1.2	12 ± 0.5	0.4 ± 0.4
Zr Reinforced GIC	16.2 ± 0.9	3 ± 0.3	0.76 ± 0.6
MTA Angelus	18.1 ± 1.1	15 ± 0.7	0.60 ± 0.5

MTA: Mineral Trioxide Aggregate.

The Tukey’s HSD multiple comparison tests found that all three experimental groups (Biodentine, zirconia reinforced GIC and MTA) had significantly lower absorbance values than the control group, and that there were no significant differences among the three experimental groups (Table 4).

**Table 4:** Pair-wise comparisons between groups.

Pair-wise comparisons	Comparison difference	95% Confidence interval		*p*
		Lower bound	Upper bound	
**Biodentine versus Zr GIC**	0.093	-0.165	0.351	0.771
**Biodentine versus MTA**	-0.038	-0.296	0.22	0.979
**Biodentine versus Control**	-0.453	-0.818	-0.088	0.010*
**Zr GIC versus MTA**	-0.132	-0.39	0.127	0.532
**Zr GIC versus Control**	-0.546	-0.912	-0.181	0.001**
**MTA versus Control**	-0.415	-0.78	-0.05	0.020*

*Source*: Original.

MTA: Mineral Trioxide Aggregate.

Tukey’s HSD multiple comparisons test. HSD: Honest significant difference (*Statistically significant,

** Highly statistically significant).

## Discussion

Perforations, regardless of their location or cause, can negatively impact the outcome of endodontic treatment. A favourable prognosis is more likely when the perforation is sealed. Immediately, whether it is iatrogenic or pathologic, delays in sealing can lead to infection of the perforation site and hinder the healing process. The prognosis of furcation perforation depends on time, size, and cause. A good prognosis is seen with small-sized perforations and immediate treatment of perforation sites, as it reduces the destructive inflammatory response of the periodontium [16]. Hence, the materials included in this study are recent materials with short setting time so that furcation perforation can be repaired as soon as possible (Biodentine – setting time 12 min, zirconia reinforced GIC – setting time 3 min, and MTA Angelus – setting time 15 min).

Microleakage is defined as the ‘diffusion of the bacteria, oral fluids, ions and molecules into the tooth and the filling material interface’ OR defined as ‘the clinically undetectable passage of bacteria, fluids, molecules or ions between tooth and the restorative or filling material’ [17]. Several methods have been used to assess microleakage through dye penetration, fluid filtration, bacterial and protein leakage models, dye extraction methods, scanning electron microscopy, and analysis with radioactive isotopes [18]. This study applied a dye extraction method. In the dye extraction method, as reported by Camps et al. [19], the actual volume of the dye absorbed is calculated by dissolution of the samples in concentrated nitric acid. The optical density of the solution was recorded using a spectrophotometer. Torabinejad et al. stated that a material that is able to prevent the penetration of small molecules like dye should be able to prevent larger substances like bacteria and their by-products [20]. This study used spectrophotometric dye extraction because of its quantitative accuracy, reproducibility, cost-effectiveness, and minimal observer bias. It allows precise comparison of microleakage through objective absorbance values, making it ideal for *in vitro* material comparison [21].

While other methods like confocal laser scanning microscopy (CLSM) offer high-resolution and three-dimensional visualisation of leakage pathways, it has limitations such as high cost, technical complexity, and subjective interpretation. It is more suitable for visual analysis rather than bulk quantification. Similarly, microcomputed tomography (micro-CT) reveals internal gaps but does not directly measure leakage and lacks sensitivity to real-time fluid or dye movement [22].

The dye used in the present study was 2% methylene blue. Oppenheimer and Rosenberg reported that the smaller sizes of methylene blue particles compared with bacteria made the dye test a more precise test than bacterial leakage models [23].

The evaluation of additional physicochemical properties, such as flow, setting time, and solubility provided valuable insight into the sealing behaviour of the tested materials.

A material that flows well even under moist conditions improves clinical success, preventing washout, ensuring intimate contact with wet dentin.

Biodentine exhibited the highest flow and the lowest solubility, suggesting excellent adaptability to cavity walls and long-term dimensional stability, contributing to its favourable sealing performance. MTA Angelus showed moderate flow, while zirconia reinforced GIC showed higher solubility, which may compromise its sealing integrity under prolonged moisture exposure.

Setting time is a critical factor when selecting a material for perforation repair, particularly in the furcation region, where blood and moisture contamination are common. A material with a shorter setting time offers several advantages: it minimises washout risk in the presence of saliva or blood, and allows immediate restoration or obturation in a single visit. Furthermore, it enhances the sealing ability, as early hardening stabilises the material before contamination can interfere [24].

Materials such as Biodentine and Zirconomer are favoured for their rapid set and moisture tolerance, compared to MTA, which has a longer setting time [25].

A material with high solubility may degrade or dissolve over time, especially in the moist environment of the furcation region, leading to loss of marginal seal and allowing bacterial and fluid infiltration. This results in dimensional instability, which compromises adaptation to dentin, reduces durability, and increases the risk of treatment failure. Materials like Biodentine and MTA exhibit lower solubility compared to conventional GICs, making them more suitable for clinical applications in perforation repair [26].

According to the study conducted by various authors, Biodentine’s ability to form tag-like structures increase its resistance to dislodgement forces. Moreover, the smaller particle size, a wider calcium and silicate-rich dentine area, and longer incorporation depths into dentine also allow for better marginal adaptation and sealing ability. Premixed form of material, which reduces the air entrapment in the mix, has a putty consistency that gives better adaptability to dentinal walls and excellent handling of the material [27].

Zirconia reinforced GIC adheres through chemical bonding with dentine and might have adequately filled or sealed the perforation. The adhesion of GICs to the tooth results from two interrelated mechanisms: micromechanical interlocking and true chemical bonding, which involves ionic bonding between the carboxyl ions in the cement and calcium ions in enamel and dentine. The micromechanical interlocking is caused by the formation of short cement tags within the surface of the dentine. Physical properties and consistency of mix (8:1 P/L) might have resulted in less solubility of zirconia reinforced GIC [28]. These favourable results could also be due to the reduced moisture interference in *in vitro* conditions versus the clinical oral environment [29].

Chaudhary et al. did a comparative evaluation of push-out bond strength of three retrograde filling materials: MTA Angelus, Zirconomer, and bioactive bone cement in teeth with root apices resected at 90°. The study stated that Zirconomer was an excellent retrograde filling material because of its superior strength and endurance, as well as chemical bonding. When compared to MTA Angelus and bioactive bone cement, Zirconomer showed higher retention [30].

Calcium hydroxide is produced as a by-product in greater quantities in MTA due to the hydration of calcium oxide. Consequently, the overall microstructure of MTA is less dense and potentially more prone to solubility and microleakage when compared to Biodentine, which forms a tighter, more homogeneous structure upon setting [31].

There are a few limitations to this study. While this study aimed to identify the most effective furcation perforation repair material among various materials, the ultimate choice also considers factors like biocompatibility, osteo-induction and osteo-conduction. Studies have shown that MTA and Biodentine possess these biologically favourable properties [32], whereas zirconia reinforced GIC lacks such characteristics. In clinical cases, the moist environment around the furcation will increase the sealing efficiency of Biodentine and MTA but can decrease the efficiency of zirconia reinforced GIC, as the perforation area is contaminated with blood and fluids [33].

## Conclusion

Within the limitations of this study, the null hypothesis was accepted, indicating that the sealing ability of Biodentine, MTA and zirconia reinforced GIC was statistically comparable.

However, further research with more samples along with the application of different techniques would be helpful. More *in vivo* research is also required to back up our preliminary findings.

## Conflicts of interest

There are no conflicts of interest.
